# Rapid Cerebral Edema and Herniation in a 65-Year-Old Man With Balamuthia Mandrillaris

**DOI:** 10.7759/cureus.14498

**Published:** 2021-04-15

**Authors:** Michael Zwillman, Anh T Nguyen, Natalie Organek, Zoulficar A Kobiessi, Sudha Kodali, Kevin E Immanuel

**Affiliations:** 1 Department of Neurosurgery, Houston Methodist Hospital, Houston, USA; 2 Section of Hepatology and Transplant Medicine, Houston Methodist Hospital, Houston, USA

**Keywords:** brain edema, amebiasis, meningoencephalitis, hypoxia brain, coma, trophozoites, non-convulsive status epilepticus, diabetes insipidus, brain herniation, hepatocellular carcinoma (hcc)

## Abstract

This case describes a 65-year-old man with hepatocellular carcinoma as well as other medical comorbidities who developed rapidly progressive cerebral edema, nonconvulsive status epilepticus, and ultimately died. Postmortem examination revealed massive cerebral edema, widespread parenchymal necrosis, herniation, hemorrhage, and cerebral amebiasis. The causative agent was identified by the Center for Disease Control as Balamuthia mandrillaris.

## Introduction

In 1986, a free-living ameba, previously thought to be non-pathogenic, was identified as the causative agent of fatal meningoencephalitis in a pregnant mandrill residing at the San Diego Zoo. This organism is similar to Acanthamoeba but different enough to establish a new genus and species [1-3]. It has now joined with the better-known causes of human amebic meningoencephalitis, Naegleria fowleri, and Acanthamoeba species. Human amebic meningoencephalitis has two pathologic disease patterns described: granulomatous amebic encephalitis and primary amebic meningoencephalitis. Naegleria fowleri causes primary amebic meningoencephalitis (PAM) and is known for rapid neurologic deterioration, secondary to hemorrhagic necrotizing meningoencephalitis, with a symptom onset one to nine days after exposure. Granulomatous amebic encephalitis (GAE) is a chronic granulomatous infectious process caused by Acanthomeba and Balamuthia mandrillaris. GAE is characterized by granular focal brain lesions leading to necrosis and edema. GAE has a chronic onset usually weeks to months from the initial infection. Even with treatment, the mortality for PAM and GAE exceeds 95% [4].

## Case presentation

**Figure 1:**
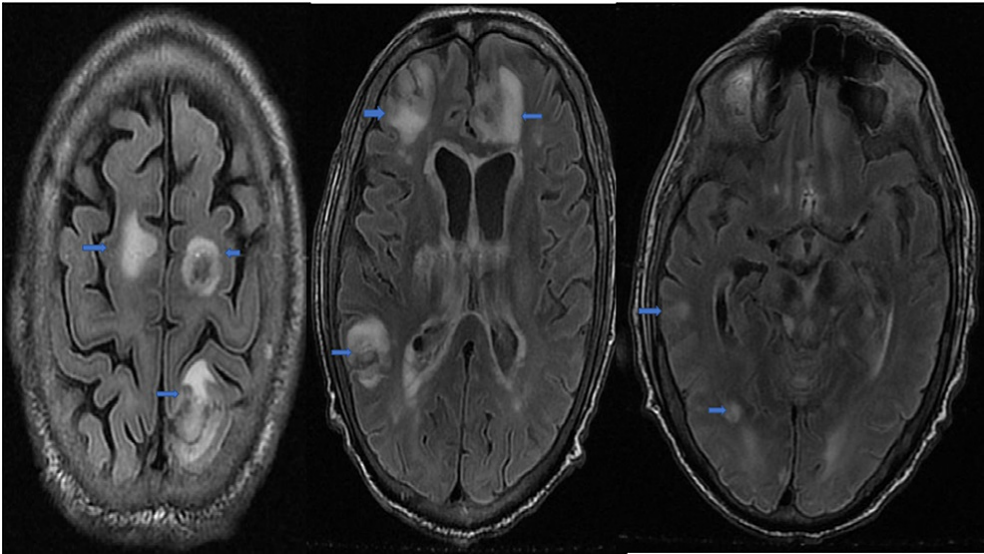
MRI head without contrast Multiple masses with hemorrhage (blue arrows)

A 65-year-old man with a past medical history significant for hepatocellular carcinoma (HCC) had recently been listed for liver transplantation. Eleven days prior to presenting to our hospital the patient presented to a medical facility near his home complaining of weakness, lethargy, lower extremity cellulitis, and fever as well as encephalopathy out of proportion to previous episodes of hepatic encephalopathy. The patient’s hospital course was significant for persistent fever, negative coronavirus disease 2019 (COVID-19), kidney stones with hydroureteronephrosis, progressive worsening encephalopathy, and airway insecurity requiring intubation and mechanical ventilation. Non-contrast CT performed at referring facility showed bifrontal left parietal and right cerebellar ischemic strokes. The patient was transferred to our hospital for a higher level of care. On the day of admission, the patient was intubated and hypotensive requiring norepinephrine pressor support. On examination, the patient was well-nourished and appeared at his stated age. He did not open his eyes spontaneously, nor to voice or pain. The sclerae were anicteric, pupils miotic but reactive to light and the gag, and corneal reflexes were present. The patient triggered the ventilator spontaneously but did not move any of his extremities voluntarily nor did he withdraw to pain. His Glasgow Coma Scale (GCS) was three. The remainder of his physical examination was unremarkable except for a sacral decubitus ulcer and healed dark plaque-like ulcers on the lower extremities bilaterally. Empiric azithromycin, ceftriaxone, vancomycin, and metronidazole were started. Transthoracic echocardiogram was unremarkable. MRI brain, magnetic resonance angiography (MRA) head and neck were obtained. The MRA of the head and neck were unremarkable. The MRI without contrast, shown in Figure 1, revealed supra and infratentorial intra-axial masses with hemorrhagic transformation, concerning metastatic disease as well as subependymal diffuse supra and infratentorial white matter hyperintensities with restricted diffusion, suggestive of ependymal carcinomatosis. The liver transplant, oncology, and neuro-oncology services were consulted given concerns for possible central nervous system metastatic disease. A lumbar puncture was considered but not done as the patient was coagulopathic. Subsequent imaging showed progressive cerebral edema with a high risk of brain herniation. Lumbar puncture was not pursued further. Continuous electroencephalography (cEEG) and levetiracetam were begun for seizure surveillance and prophylaxis, respectively. Decadron was initiated to decrease vasogenic edema. Initial labs were concerning for anemia, leukopenia, thrombocytopenia, hypoalbuminemia, coagulopathy, and were attributed to existing hepatocellular carcinoma. 

On the second hospital day, the patient remained encephalopathic despite no sedatives or analgesics. No seizure activity was recorded, but the neurologic examination worsened; the pupillary light reflex on the left was slow and absent on the right. The previously present gag reflex was not detected. The patient remained comatose, GCS score was three. A CT scan of the chest, abdomen, and pelvis without contrast was obtained for primary cancer and metastatic assessment, which were unremarkable. Abdominal imaging revealed a single, small lesion consistent with his known history of hepatocellular carcinoma and unchanged from recent imaging obtained for transplant workup. The alpha-fetoprotein levels were within normal limits making the present neurologic findings unlikely to be secondary to liver metastasis.

On the third hospital day, serology for Epstein Bar virus was positive. Acyclovir was added for antiviral therapy and fluconazole added for fungal prophylaxis, respectively. Human T cell lymphotropic virus I and II were negative but the absolute cluster of differentiation 4 (CD4) count was 123 per microliter (488-1340 per microliter). The infectious disease team felt the low CD4 state cause by his existing liver disease. West Nile virus exposure was demonstrated with positive immunoglobulin G (IgG), but immunoglobulin M (IgM) was negative. Cytomegalovirus (CMV) was not detected but reported postmortem. Coccidiodes IgG and IgM were negative. Histoplasma and Legionella antigens were negative. Strongyloides antibody was positive but felt not significant to his current illness. Doxycycline was added for atypical organisms and tick-borne illnesses. 

On the fourth hospital day, blood cultures were positive for vancomycin-sensitive Staphylococcus epidermidis. These were the only cultures positive throughout hospitalization. Due to the low CD4 count, atavaquone was added for prophylaxis against opportunistic infections. Seizures were detected on cEEG. Urine output, monitored hourly, suddenly increased concerning diabetes insipidus (DI). The sodium level peaked at 169 mEq/L and was corrected over three days using intravenous vasopressin and 5% dextrose solution. The seizures were treated with antiepileptics.

**Figure 2:**
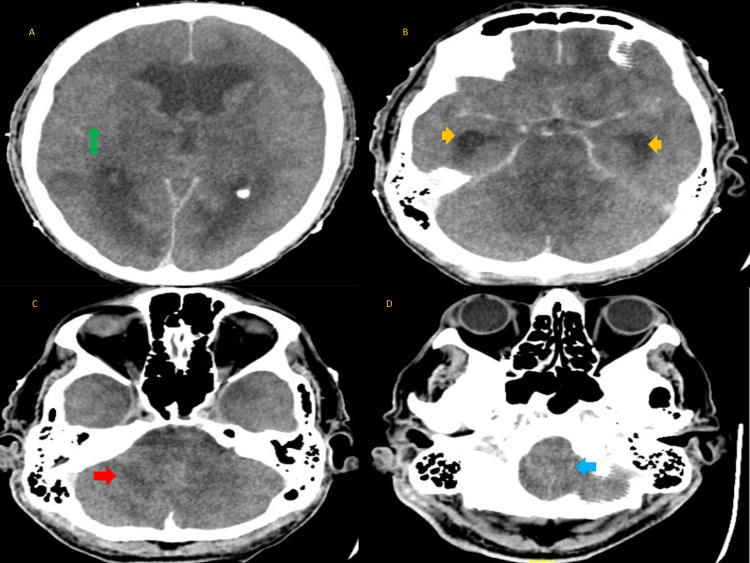
CT head with contrast Anoxic injury:  (A) diffuse cerebral edema loss of sulci and gyri (green arrow), (B) hydrocephalus (yellow arrows), (C)  cerebellar edema (red arrow), and (D) herniation (blue arrow).

On the fifth hospital day, the patient remains comatose but was no longer making a ventilatory effort, indicative of deteriorating neurologic status. A contrast CT scan of the head was obtained (Figure 2).

It showed anoxic brain injury from diffuse edema involving the cerebral hemispheres, cerebellum, and brain stem. A CT angiogram (CTA) of the brain showed no opacification of cerebral vessels. The patient’s family elected to withdraw life support and he was pronounced dead on the hospital seventh day. The family also consented to a postmortem examination. 

**Figure 3:**
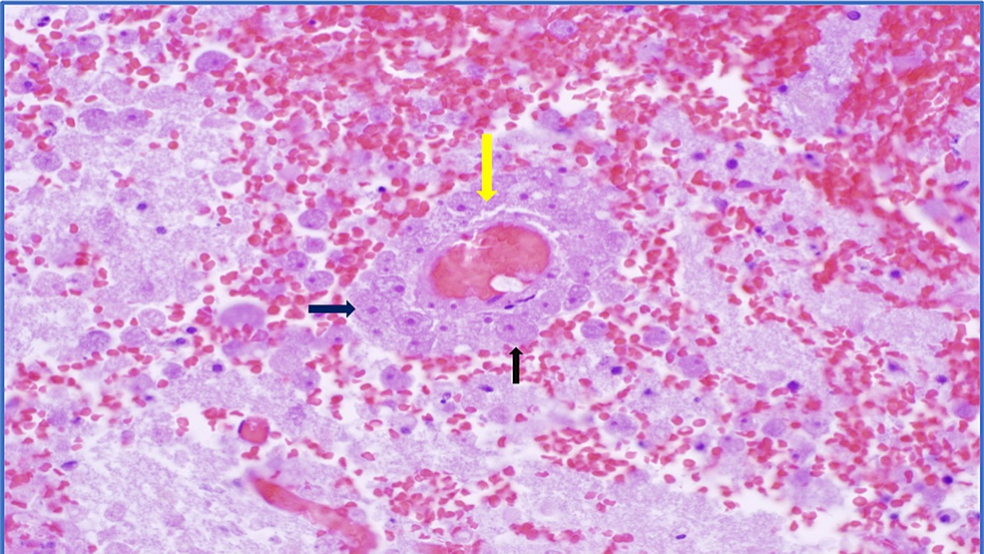
Hematoxylin and eosin staining of infarcted brain tissue Suspected trophozoites (black arrows) surrounding a capillary (yellow arrow)

The postmortem examination was performed on the ninth hospital day. Gross examination of the brain revealed massive cerebral edema, widespread parenchymal necrosis, herniation, and hemorrhage. The cerebellar tonsils are tightly opposed to the surrounding brainstem compatible with tonsillar herniation. On microscopic examination almost all brain areas showed infarcted parenchyma with necrosis, fibrin thrombi, and hemorrhage. Trophozoites with abundant granular cytoplasm and prominent nucleoli were seen in the vast majority of tissue sections. The structures are present in the perivascular region as well as in the neuropil and around small capillaries (Figure 3).

**Figure 4:**
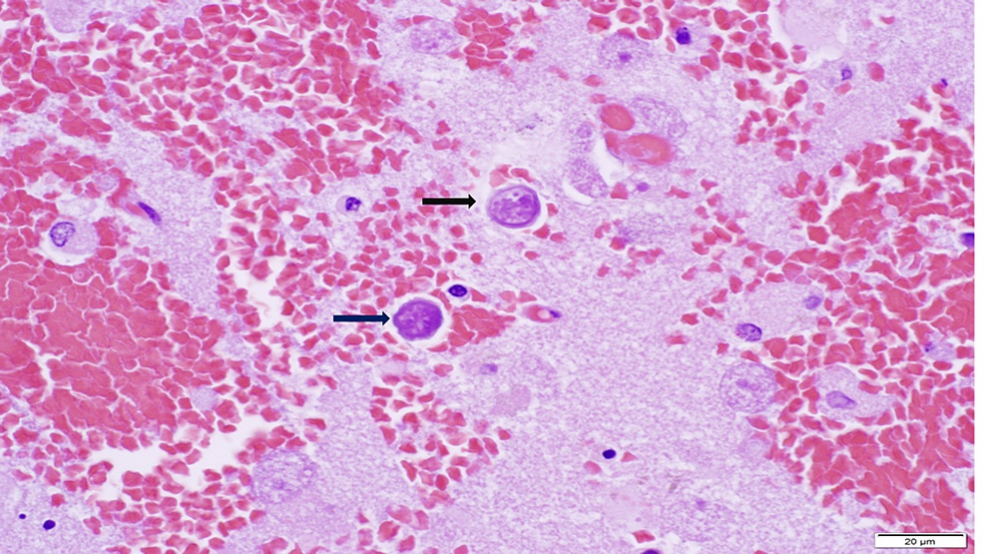
Hematoxylin and eosin staining of brain tissue Cysts (black arrows)

There was no granulomatous inflammation in any tissue sections. Thick-walled encysted forms were seen which was diagnostic of amebiasis (Figure 4). Tissue samples were sent to the Center for Disease Control Infectious Diseases Pathology Branch for further evaluation. Histopathologic studies confirmed the presence of trophozoites. Immunohistochemical assays were performed staining most intensely for Balamuthia but there was cross-reactivity with Acanthamoeba. Final and definitive identification was performed using real-time polymerase chain reaction confirming Balamuthia mandrillaris.

## Discussion

Balamuthia mandrillaris is the only known species of this genus that causes both animal and human disease. It exists as a trophozoite or cyst, with the trophozoite being the infective form. Entry into the body is thought to occur by inhalation of cysts or direct contamination through skin defects. The organism spread is via the hematogenous route. Balamuthia lives in soil with worldwide distribution. Initially, it was thought to live exclusively in warm and dry climates, but this was proven untrue. Balamuthia infection was thought to affect only the extremes of age and immunocompromised individuals; this too has been disproven as immunocompetent children and adolescents have been affected [5]. A review of the literature shows that the infection affects infants to older adults with cases reported in patients from four months to 91 years (median age: 36 years), 68% of these were males. In those with known ethnicity, 55% were Hispanic. In those patients with a documented outcome, 90% died [6]. There have been approximately 200 cases worldwide with 109 of these documented in the United States, especially in California, Texas, and Arizona. The patient described in our case was a 65-year-old Hispanic male, who prior to his death, resided in southern Texas. Cerebrospinal fluid in GAE caused by Acanthomeba and Balamuthia shows lymphocytosis, elevated protein, and normal to low glucose concentrations. Trophozoites may be sparse or few on CSF wet mounts. Cysts are not usually seen. CSF analysis is not the sample of choice to rule out GAE. Histopathologic examination of brain tissue has the highest yield of diagnosing trophozoite infection [7].

In terms of treatment, CDC recommends a combination of flucytosine, pentamidine, fluconazole, and either azithromycin or clarithromycin. There has been some success with the use of this regimen plus miltefosine [8]. Further evaluation is required to determine a treatment standard.

## Conclusions

Human amebic meningoencephalitis is a rare and underdiagnosed disease with high morbidity and mortality and should be in the differential for new-onset encephalitis especially in patients from California, Texas, and Arizona. Although the course of the illness is generally gradual progressing over weeks to months, to our knowledge, this is the first case in which radiographic and clinical brain herniation occurred. Early diagnosis, clinical suspicion, and tissue pathology are paramount for patient survival.
